# Defense Mechanisms Reloaded in the Light of Impaired Personality Functioning: An Attempt of Clarification and Simplification Resulting in the DSQ-22-A for Adolescents

**DOI:** 10.3389/fpsyt.2022.866837

**Published:** 2022-05-27

**Authors:** Lea Sarrar, Kirstin Goth

**Affiliations:** ^1^Department of Psychology, Faculty of Sciences, Medical School Berlin (MSB), Berlin, Germany; ^2^Department of Child and Adolescent Psychiatry, Psychiatric University Clinics Base, Basel, Switzerland

**Keywords:** Defense Style Questionnaire, defense mechanisms, personality functioning, adolescents, psychometric properties

## Abstract

With the upcoming ICD-11, the diagnostic guidelines for personality disorders will change fundamentally to a dimensional severity concept, including the evaluation of several domains of personality functioning. Moreover, the lifetime perspective will allow this diagnosis even in early adolescence, providing the opportunity for early detection and intervention. In psychodynamic understanding, defense mechanisms are considered to be a part of the “personality structure”, which is one axis in the related diagnostic system (OPD) and showed great similarities to the concept of personality functioning. The most common inventory to assess defense mechanisms is the Defense Style Questionnaire, especially the DSQ-40, which has unfortunately not been specifically adapted to younger ages yet. Using an age-adapted version of the DSQ-40 with simplified formulations, a thorough empirical item analyses and selection was performed, including a face-validity check of the items by experienced therapists and assessments for item correlations, factor structure, reliability, construct and clinical validity in a german clinical and school sample containing 396 adolescents. Though several improvements, similar problems as reported for the adult DSQ versions concerning face-validity and coherence of the item pairs (2-item-method) to represent the single defense mechanisms were obtained. Thus, not all item pairs could be kept and a shortened version DSQ-22-A for adolescents with good psychometric properties was build. The three resulting defense categories adaptive, neurotic and maladaptive showed acceptable scale reliabilities (0.63, 0.56, 0.68), sound factor structure and convincing convergent and clinical validity in terms of highly significant correlations with impaired personality structure according to the OPD-CA2-SQ as well as with PHQ-D depression and somatic symptoms, especially for the maladaptive defense category (0.75, 0.44, 0.34). Likewise, the maladaptive defense category differed highly significant (*p* = 0.000) and with a large effect size of *d* = 0.9 standard deviations between adolescents from the school and the clinical sample. The *DSQ-22-A* can be recommended for use in adolescents for research, diagnostics and therapy planning, especially with regard to personality functioning and structure. Possible fundamental changes concerning some basic operationalization's of the defense mechanisms and the 2-item-method were suggested for international discussion.

## Introduction

The definition and diagnosis of personality disorders (PD) is currently in a state of transition (1, 2). With the upcoming 11th version of the International Statistical Classification of Diseases and Related Health Problems [ICD-11; (3)] diagnostic guidelines for PD will change fundamentally. PD are then viewed as a continuum of “no” to “severe” impairment in basic levels of functioning, expressed by a summary severity score independent from any former type of PD. Whether impairment is present is assessed on the basis of problems in functioning related to aspects of self and interpersonal dysfunction, further weighted with regard to specific socio-psychological aspects. The changes correspond to the Alternative Model of Personality Disorders (AMPD) in the research section of the 5th version of the Diagnostic and Statistical Manual of Mental Disorders [DSM-5; (4)], recommending a dimensional diagnostic of PD by using a severity approach (Criterion A). In addition, the age restriction for PD is abolished in the ICD-11 (3) following a general lifetime perspective and a unified system of psychopathology for all ages. Thus, the diagnose PD can be assigned even in early adolescence, provided that the criteria are fully met. Clinicians and researchers, in support of an early diagnosis of PD, argue that early detection and early intervention are crucial to prevent severe impairment of the developing personality. They also argue in favor of dimensional models of PD to identify at-risk individuals who may profit from interventions that lead back to a path of less severely impaired, respectively healthy personality development (5).

There is also a psychodynamically based multiaxial diagnostic classification system for adults [Operationalized Psychodynamic Diagnosis System; OPD; (6)] and – in an age-specific version – for children children and adolescents [OPD-CA-2; (7)] that complements symptom-oriented diagnostics according to DSM or ICD. Within the OPD-CA-2 (7), four axes (treatment demands, relationships, conflicts and structure) are described. The axis personality structure is especially related to PD and contains four different domains (control, identity, interpersonality and attachment) which correspond exceedingly clearly to the concept of personality functioning denoted in the DSM-5 AMPD (with the domains identity, self-direction, empathy and intimacy) to describe the core impairments common to all PD (4, 7). Personality structure is defined as the availability of psychological functions to regulate the self and its relation to internal and external objects (6, 7). This refers to the acquired tools for regulating and processing conflict situations, stresses and strains or development tasks. A careful diagnosis of structural difficulties and competences is indispensable for indication and therapy planning within the psychotherapeutic practice. The assessment of the level of personality structure is usually done by trained OPD experts using an OPD interview (6, 7).

Empirical findings using the OPD-CA-2 interview (7) indicated that adolescents with mental disorders show deficits in personality structure (8–10). However, the use of these interviews require a large amount of time, limiting the possibility to be used in research. Thus, OPD Structure Questionnaires [for adults OPD-SQ (11); for adolescents OPD-CA2-SQ (12)] were developed to assess personality structure in self report. Recent studies with the OPD-CA2-SQ (12) supported the applicability of the questionnaire in adolescents from school (13, 14) as well as clinical samples (15). Schrobildgen et al. (15) reported for the OPD-CA2-SQ (12) a highly significant discrimination between school population and patients with PD using the total score with a large effect size of *d* = 1.6 standard deviations (see below) and – compared to that – a likewise discrimination of students and patients with PD according to the Levels of Personality Functioning Questionnaire [LoPF-Q 12-18; (16)] at a very similar level with a very large effect size of *d* = 2.1 standard deviations. Thus, it could be argued that the structural concept according to OPD is nearly equivalent to the personality functioning concept according to the DSM-5 AMPD (4) and ICD-11 (3) in terms of clinical utility, as with both specifically derived self-report questionnaires a valid clinical discrimination could be reached.

Defense mechanisms (DM) are another central psychodynamic concept and are considered to be fundamental to the organization and functioning of personality (6, 7, 17). They can be understood as part of the personality structure and it is assumed that a low integrated structural level is associated with the use of immature DM (7). The description of DM is based on the assumptions of A. Freud (18). They are considered to be an unconscious ego function, used to protect the conscious mind from feelings of anxiety (19). Vaillant (20) proposed a model of ego defense in which the DM can be arranged on a continuum of ego maturity from mature to immature, judged by their flexibility, functionality, variability, continuity as well as reality distortion. Even though it is postulated that DMs function unconsciously, there is evidence that people may be aware of certain parts (21).

There had been attempts to provide conclusive models on how many relevant DM exist [DSM-IV; (6, 7, 17)] and efforts to quantitatively measure those, which has led to the development of several versions of self-report inventories gathered under the name Defense Style Questionnaire (DSQ). After an extensive literature search (for a selection of DSQ studies see supplement), we found 12 different versions of the DSQ (as far as access was possible). However, many of these versions are not well described, so we will focus on the most essential ones in the following.

Initially, an 81-item version of the DSQ assessing 24 different DM and providing four higher-order defense categories — based on an exploratory factor analysis — was introduced by Bond et al. (22) with the categories: adaptive (mature), image-distorting, self-sacrificing (neurotic) and maladaptive (immature). Bond et al. (23) modified the 81-item version by changing to a 2-item system per DM and published the according 88-item version, also providing the above mentioned 4-factor solution. From then on, the DSQ was revised several times, both in terms of the number of items, the content of the items and the factor structure. For example, a 72-item DSM-lll-R-labeled version of the DSQ (24) was developed with a 3-factor solution, interpreted as mature, neurotic and immature defense category.

Finally, the most common and prominent 40-item version of the DSQ (25) was published. Following the DSQ-88 (23), a 2 item system assessing now 20 DM using a 9-point Likert-type scale with three higher-order defense categories (mature: α = 0.68, neurotic: α = 0.58, immature: α = 0.80) was chosen. Andrews et al. (25) showed that the DSQ-40 was able to discriminate significantly between patient (anxiety disorders, child-abusing parents) and healthy samples (*p* < 0.05). Today, the DSQ-40 is a widely used self-assessment tool that has been translated into numerous languages [e.g., French: (26); Italian: (27); German: (28)]. Nevertheless, the DSQ-40 has some psychometric difficulties, as shown by various studies. Schauenburg et al. (28) reported insufficient pairwise intercorrelations for some items. Chabrol et al. (29) and Schauenburg et al. (28) revealed unsatisfactory face validity of the original English and the German version of the DSQ-40 and, therefore, deleted several items. Both studies (28, 29) reported, in agreement with the original DSQ-40 version (25), a 3-factor solution.

Also modified DSQ versions for adolescence exist. In particular, the research group around Steiner et al. has been very active in this regard. In several studies (30–33), they used a 78-item modification of Bond's original DSQ-81 (22), in which 19 DM were assessed by one to nine items on a 9-point scale in adolescents 12 years of age and older. Unfortunately, the item modifications were not described in more detail. However, the studies showed that the DSQ concept is applicable in principle for adolescents. Comparable to Andrews et al. (25), but in contrast to Bond et al. (22), Steiner and Feldman (31) found a 3-factor solution matching the defense categories mature (α = 0.52), neurotic (no statement of α) and immature (α = 0.81) category. Some of the category scores were able to discriminate significantly between healthy samples and specific pathological groups (immature and mature defense category for boys between a delinquent and healthy sample with *p* < 0.01; neurotic and mature defense category for girls between a psychosomatic and healthy sample with *p* < 0.05). Subsequently, there have been attempts (34, 35) at shortening the adolescent version of the DSQ (30). However, none of the youth-specific versions has been widely popular.

In several studies (21, 36) the original DSQ-40 version for adults (25) had been used in adolescent samples, starting at ages 10 (36) and 13 years, respectively (21). Ruuttu et al. (21) used a Finnish translation of the DSQ-40 and found a 4-factor solution explaining 49% of the total variance with mature (α = 0.62), image-distorting (α = 0.62), neurotic (α = 0.60) and immature (α = 0.78) defense category. Furthermore, they demonstrated that all four scales were able to discriminate significantly between an adolescent healthy and patient sample with mood disorders (immature, image-distorting and mature with *p* < 0.001, neurotic with *p* = 0.002). Moreover, they reported reasonable and significant correlations between the DM categories and psychiatric symptoms for immature (0.65), neurotic (0.15), image-distorting (0.20) and mature (−0.45). Likewise, reasonable correlations with adaption for the category immature (−0.54), neurotic (−0.10), image-distorting (−0.25) and mature (−0.35). In addition, they postulated to have confirmed the face validity of the original DSQ-40 (25) in adolescent samples, however, without giving any further details. Another study (36) used a Greek translation of the DSQ-40 and also postulated satisfactory internal consistency for four defense categories (mature: α = 0.58, image-distorting: α = 0.61, neurotic: α = 0.60, immature α = 0.75,). Moreover, they assumed that the DSQ-40 is a valid instrument for use in childhood and adolescence. They indicated the construct validity in terms of significant intercorrelations between each of the 4 defense categories with ranges from 0.20 (image-distorting and neurotic category) to 0.62 (immature and image-distorting category). Convergent validity was reported by e.g., significant positive associations between mature (0.20) and significant negative associations between immature defense category (−0.22) and psychological wellbeing, while neurotic defense category (0.17) correlated positive with psychological wellbeing, thus, not corresponding to the assumed relation. Moreover they reported, that the immature defense category predicted psychological wellbeing, bullying behavior, as well as the experience of victimization (*p* < 0.001; *OR* = 0.95–1.07), the neurotic defense category predicted psychological well-being (*p* < 0.05; *OR* = 1.05) as well as bullying behavior (*p* < 0.01; *OR* = 0.96), and finally, the mature defense category predicted psychological well-being (*p* < 0.01; *OR* = 1.06) as well as the experience of victimization (*p* < 0.001; *OR* = 0.09).

The current body of research suggests that while the defense concept can be usefully applied in adolescence, there is no age-specific version of the most current and widely used DSQ inventory for adolescents: the DSQ-40 (25). In addition, it can be noted that the problems with face validity and psychometric properties have not been resolved for any of the current DSQ-40 versions.

In the light of the new dimensional assessment of PD also in younger ages and the obvious suitability of psychodynamic concepts in this context, the consideration of DM seems to be highly topic. DM, despite the ambiguities and partly problematic quality criteria of previous operationalization's as questionnaires, showed clear relationships to psychopathology. In order to be able to investigate the possibility that DM can elucidate specific pathological developments in adolescence, the first step requires the development of a reliable and valid assessment tool for that age group. Thus, the present study aims to take a close look at the concept of DM based on the most recent operationalization for adults and to develop an optimized and age-specific assessment tool for adolescents from 12 years up (+/− 2 years).

### Development of an Age-Specific Research Version DSQ-40-A for Adolescent Self-Report

In consultation with Mr. Schauenburg, a specific version with adapted wording for adolescents was developed on the basis of the German version DSQ-40 for adults (28). Almost all items were slightly changed and simplified in order to be easier to understand and to better fit into the everyday world of young people. In doing so, we drew on the extensive experience with age-adapted test construction gained both in the development of the Junior Temperament and Character Inventory test family [JTCI-R; (37)] with content-equivalent test versions for 3–6 year-olds, 7–11 year-olds, 12–18 year-olds and adults to elicit Cloninger's personality model and in the development of youth-adapted assessments of the level of personality functioning concept [LoPF-Q 12–18; (12)]. The main goal was easy linguistic comprehensibility, unambiguity of the core content and avoidance of socially desirable responses (e.g., by leaving out terms or examples that young people typically find “embarrassing”). A special attempt was made to find new formulations for those items that had shown very unsatisfactory face validity and reliability in the original DSQ-40 adult versions (28, 29) in order to not lose the affected DM “Deevaluation”, “Dissociation” and “Displacement” in the new test version. **Table 2** shows all item formulations of the adult version and the youth version in comparison.

In our view, three items in particular have undergone major changes in wording that are intended to sharpen the core of the concept and thus may have changed it somewhat (see Table 1).

**Table 1:** Examples and explanations for major changes of item formulations from adult to adolescent version of DSQ.

**Original for adults**	**Changed for adolescents**	**Content**
**Item 1**:		
“I get satisfaction from helping others and if this were taken away from me I would get depressed.” (29) “It is important for me to help others. When I am no longer able to do that, I get depressed.” [(28), translated from German into English)	Leaving out the signal word “depression” to avoid pathological misinterpretiation. Avoiding two seperated sentences. “It is so fulfilling and important for me to help others that I need such a task in my life.”	Formulated to capture the neurotic defense mechanism “Pseudoaltruism”.
**Item 9**:		
“I ignore danger as if I was Superman.” (29) “I don't pay attention to danger as if I was invulnerable.” [(28), translated from German into English]	Leaving out the signal word “invulnerable” or “superman” to avoid socially desired responses. “I rather don't pay attention to dangers because I have a strong and secure feeling that nothing will happen to me.‘”	Formulated to capture the maladaptive defense mechanism “Dissociation”. Whether this item content fits the concept of Dissociation could be discussed (Glossary DSM-IV: individual deals with emotional conflict or internal or external stressors with a breakdown in the usually integrated functions of consciousness, memory, perception of self or the environment, or sensory/motor behavior)
**Item 16**:		
“There are always good reasons when things don't work out for me.” (29) “I always find excuses when something doesn't go well.” [(28), translated from German into English]	Leaving out the negative phrase “finding excuses” to sharpen the content where “saying yes” clearly stands for a healthy and mature defense mechanism. “If something is not going well in my life, I analyze exactly what the reasons are until I understand it.”	Formulated to capture the adaptive defense mechanism “Rationalization”. The phrase “finding excuses” has a negative connotation. This probably does not fit to the targeted content “positive mature defense”

Using this German age-specific research version of the DSQ-40 for adolescents, the validation steps that had been taken for the adult versions had been replicated and extended in order to investigate the possibility of a reliable and valid assessment of the 20 DM in adolescence. The main focus lied on the clinical validity–especially on the relation with impairments in personality structure according to the OPD-CA2-SQ (12) – in order to incorporate a tool directly relatable to personality functioning (Criterion A) as an external criterion of validity.

## Materials and Methods

### Participants and Procedure

#### Clinical and School Sample

The clinical sample consists of 135 psychotherapeutic patients of six youth care facilities, four psychotherapeutic practices as well as a clinic for child and adolescent psychiatry (*M*_*age*_ = 17.8; *SD* = 1.7; 67% female, 30% male and 3% diverse sex). These patients were recruited since 2020. Inclusion criteria was the existence of a clinical diagnose according to the Patient Health Questionnaire [PHQ-D; (38)]. 55.9% of the adolescents met the criteria for only one syndrome. Among these, alcohol-related syndromes were found in 40.0%, depressive in 36.2%, somatoform in 16.3%, anxiety-related in 6.2% and eating disorder syndromes in 1.3% of the adolescents. Two syndromes were present in 27.3% of the adolescents, three in 15.4% and four or five each in 0.7%. Within the patient group there were significant differences regarding sex [χ^2^(2) = 89.47, *p* < 0.001].

The school sample consists of 261 adolescents (*M*_*age*_ = 17.2; *SD* = 2.1; 57% female, 42% male and 1% diverse sex) assessed at four schools and five out-of-school facilities since 2019. Initially, in-person group testing was conducted. Since the start of the Covid pandemic, participants completed questionnaires at home and surveys were conducted by post (since March 2020). Inclusion criterion for the control group was the presence of mental health according to PHQ-D (38) in terms of no syndrome being fulfilled. Significant differences in sex also emerged within the school sample [χ^2^(2) = 136.44, *p* < 0.001].

The total combined sample is composed of 396 adolescents (*M*_*age*_ = 17.4; *SD* = 2.0; range 12–21; 58,7% female, 39,7% male and 1,5% diverse sex) and reported very low (9.1%/6.1%), low (14.7%/13.7%), medium (33.6%/36.1%) or high (37.1%/35.0%) socioeconomic status. 8 adolescents in the clinical sample and 13 adolescents in the school sample did not report socioeconomic status. There were no statistically significant differences in socioeconomic status between the two groups. However, both groups differed significantly with respect to age [*t*(343.49) = 3.32, *p* = 0.001].

All participants were informed about the use of their data and the compliance with data protection regulations. All participants provided written informed consent. For adolescents under the age of 16, the written consent was also given by their legal guardians. The project was approved by the Research Ethic Board at the MSB Medical School Berlin (approval number: MSB-2020/30).

#### Expert Sample

Analogous to the study design of Chabrol et al. (29) and Schauenburg et al. (28), the first step was to check the face validity of each item in an expert test by assessing the degree of agreement among analytically oriented clinicians as to which DM an item is supposed to be representative for. Similar to Chabrol's et al. (29) approach, experts were presented with a combined list of the 30 DM and coping styles listed in the DSM-IV (17) and OPD-CA-2 (7) in order to facilitate attribution. However, raters were informed that also other categories than those listed could be attributed. In addition, it was asked to indicate for each item which level of maturity is represented (adaptive, neurotic, maladaptive) in order to be able to analyze this higher-level aspect of construct validity as well. The group of psychoanalytic or psychodynamic oriented experts was composed of five women and five men, most of them working at university clinics in Germany or Switzerland, seven of them were therapists with over 10 years of work experience. The list of all 40 age-adapted DSQ-40-A items were given in running order as a table without explaining that each mechanism was represented by only two items or giving information on the underlying DSQ concept.

### Measures

The *DSQ-40-A pilot version* is an age-adapted version of the DSQ-40 for adults in german language introduced by Schauenburg et al. (28). It was designed to assess Defense Mechanisms (DM) in adolescents from 12 to 18 years (+/- 2 years) in self-report following the approach of the original authors of the DSQ-40 (25). Accordingly, 20 DM, represented by two items each, are assessed on a 9-point Likert scale varying from 0 = “not true” at all to 8 = “completely true”. Based on factor analytic results, three higher-order categories are formed from the 20 DM, which correspond in content to the three different maturity categories adaptive, neurotic and maladaptive. In the current study, the psychometric properties of this pilot version are analyzed. Our aim is to create a reliable and valid final version of the DSQ-A based on empirical item selection.

The *OPD-CA2-SQ* (12) is a self-report questionnaire for adolescents between 12 and 18 years (+/- 2 years) to assess the dimensions of personality structure: control, identity, interpersonality and attachment. The development was based on the descriptions of the axis “structure” in the multiaxial diagnostic and classification system OPD-CA-2 (7). The concept of structure is similar to the new dimensional severity approach in the DSM-5 (4) and ICD-11 (3) to describe PD in terms of impairments in personality functioning, varying from a healthy to an impaired functioning. The test contains 81 items with a 5-step answering format (0 = “no” to 4 = “yes”), high scores suggest a high level of impairment. The four resulting primary scales are each composed of several subscales, matching the OPD-CA-2 (7) concept. A total score is obtained from all items to quantify a general severity level of structural impairment. Good scale reliabilities are reported with Cronbach's alpha 0.98 on total, 0.91, 0.93, 0.87 and 0.90 on primary and 0.61 to 0.85 on subscale level. Good clinical validity is reported with the total score differentiating between adolescents from a general population and a subsample of *n* = 70 patients diagnosed with PD at a highly significant level and with a large effect size of *d* = 1.6 standard deviations. The test can be requested for free for research purposes and is also available in electronic format at the project website (academic-tests.com).

The *PHQ-D* (38) detects the presence of most common mental disorders on syndrome levels. Based on 58 items, 16 diseases in five different categories (somatoform, depressive, anxiety, eating and alcohol-related disorders) are assessed. Even if the PHQ-D (38) is not capable of capturing all the information necessary for a complete diagnosis, the instrument has proven to be feasible in terms of the screening of mental disorders in previous studies [e.g., (39)]. Psychometric analyses by Gräfe et al. (40) demonstrated a high level of construct and criterion validity. The calculation of internal consistencies is considered useless, as these are only evaluated categorically and with specified jump rules [see (40)].

### Data Analytic Strategy

We used SPSS 26 for statistical analyses. In order to be able to compare the results directly, the same methods and criteria were used as in the studies on the adult versions DSQ-40 by Chabrol et al. (29) and Schauenburg et al. (28).

For each item it was counted how often a) the mechanism and b) the maturity category were correctly assigned by the experts. Based on the criteria in Chabrol et al. (29) and Schauenburg et al. (28), the face validity should be at least 40%, 70% correct classification should be aimed for. Pearson correlations were calculated for the item pairs per mechanism as indicators of shared content and reliability, following the 2-item method of the original authors. Going beyond the criteria of Schauenburg et al. (28), the correlations should be not only significant but highly significant (0.01% level) and around 0.30 or higher in order to denote a substantial relationship (medium effect size *r* > 0.30). Accordingly, factor loadings should be at least > 0.30 (better > 0.40) on the theoretically assigned factor (adaptive, neurotic, maladaptive) in Exploratory Factor Analyses (PCA, Varimax rotation) restricted to three components, matching Schauenburgs et al. (28) approach. In order to set an additional and new focus in the assessment of the quality and the final selection of the items, the reference to pathology was directly included. Each item was supposed to ideally have a significant correlation with the total score of structural impairments [OPD-CA2-SQ; (12)] or with the score for depression or somatic symptoms [PHQ-D; (38)] in order to meet the actual purpose of assessing DM in the context of e.g., expert opinions or therapy planning, namely to derive a reference to pathological or healthy behavior.

The final selection of item pairs was based on a synopsis of all the above criteria. The resulting scales were analyzed concerning their scale reliability Cronbach's Alpha, their convergent and clinical validity by Pearson correlations with all scales and subscales of personality structure and pathology as well as their potential to discriminate between adolescents with and without pathology according to the PHQ-D (38), evaluated by using the effect size measure of Cohen's *d*.

## Results

### Face Validity of the Items in the Expert Test

To enable a thorough discussion on construct validity, Table 2 shows the formulations and detailed results for all items in contrast to the original formulations for adults. To facilitate result interpretation, mean correct attributions of the items to the DM and categories by the experts are reported in the following.

**Table 2:** DSQ 40 item formulations for adolescents and results for (A) face validity by expert assignments (*N*= 10, therapists) and for (B) construct validity and reliability by empirical survey (*N* = 403, adolescents). Comparison to the original item formulations for adults (28) and to results of expert assignments for adults (28, 29).

**2.1 For the defense mechanisms of the adaptive defense category**.							
**Item**	**Defense mechanism/item formulation**	**Face validity/correct** **expert assignment**	**Correlation with patholog**			**r**_**inter**_	**f**_**mal**_
			**Structure**	**Depress**	**Somat**		
X	1. Anticipation					0.12^*^	0.36
30	Adult: same	✓					
	Adolesc.: When I have to face a difficult situation I try to imagine what it will be like and plan ways to cope with it.	7/10 DM - 9/10 CAT					
35	Adult: same	✓					
	Adolesc.: If I can foresee that things are going to get bad for me, I can deal with it better.	10/10 DM - 9/10 CAT					
✓	**2. Humor**					0.37^***^	0.60
4	Adult: It's not hard for me to laugh at myself.	✓					
	Adolesc.: It's easy for me to laugh at myself.	9/10 DM - 10/10 CAT	−0.25^***^	−0.17^***^			
26	Adult: same	✓					
	Adolesc.: I can usually also see the funny side of an otherwise painful situation.	8/10 DM - 8/10 CAT	−0.10^*^				
X	3. Rationalization					0.45^***^	0.29
5	Adult: I am able to find good reasons for everything I do.	✓					
	Adolesc.: I try to be clear about the reasons for everything I do.	9/10 DM - 3/10 CAT					
16	Adult: I always find excuses when something doesn't go well.	✓					
	Adolesc.: If something is not going well in my life, I analyze exactly what the reasons are until I understand it.	10/10 DM - 3/10 CAT					
✓	**4. Sublimation**					0.33^***^	0.49
3	Adult: I work out my anxiety through doing something constructive and creative like painting or woodwork.	✓					
	Adolesc.: I get rid of bad feelings like sadness or anxiety by doing something creative or meaningful (like painting, sports, music, fixing something).	7/10 DM - 8/10 CAT	−0.28^***^	−0.26^***^	−0.20^***^		
38	Adult: Sticking to my current task helps me to keep away from feelings of sadness or anxiety.	✓					
	Adolesc.: If I focus on my tasks, it helps me against feelings of sadness or anxiety.	3/10 DM - 5/10 CAT		−0.15^**^			
✓	**5. Suppression**					0.44^***^	0.60
2	Adult:I'm able to keep a problem out of my mind until I have time to deal with it.	✓					
	Adolesc.: I can block out my worries and problems when I have something important to do until I have time to deal with it.	6/10 DM - 7/10 CAT	−0.22^***^	−0.15^**^			
25	Adult: I can put my feelings on the back burner if they would hinder me in my current activity.	✓					
	Adolesc.: I can suppress my feelings if they would interrupt or hinder what I am currently doing.	5/10 DM - 2/10 CAT					
**2.2 For the defense mechanisms of the neurotic defense category**.							
X	**6. Acting Out**					0.32^***^	0.05
11	Adult: I often act quite suddenly and impulsively when something worries me.	✓					
	Adolesc: When something worries me a lot or burdens me, I often act quite suddenly and without thinking.	7/10 DM−2/10 CAT	0.34^***^	0.18^***^			
20	Adult: I get openly angry and aggressive when I feel hurt.	✓					
	Adolesc: When I feel hurt or offended, I also directly do something hurtful or aggressive.	7/10 DM−3/10 CAT	0.36^***^	0.17^***^	0.10^*^		
X	7. Pseudoaltruism					0.21^***^	0.68
39	Adult: If I were in a crisis, I would talk to someone who had a similar problem.	1/8 (Chabrol) Elimin. (Sch.)					
	Adolesc: If I were in a crisis, I would look for people who have similar problems to find solutions together and let others benefit from my experience.	3/10 DM−2/10 CAT					
1	Adult: It is important for me to help others. When I am no longer able to do that, I get depressed.	✓					
	Adolesc: It is so fulfilling and important for me to help others that I need such a task in my life.	10/10 DM−6/10 CAT					
✓	**8. Idealization**					0.24^***^	0.36
21	Adult: I always know someone who I see as flawless and ideal.	✓					
	Adolesc: I have often had the feeling that someone I know is like a guardian angel.	8/10 DM−6/10 CAT	0.12^*^				
24	Adult: I know someone who can achieve anything and who is 100% fair and just.	✓					
	Adolesc: I know someone who I am sure can achieve anything and is 100% fair and just.	8/10 DM−5/10 CAT					
✓	**9. Reactive Formation**					0.24^***^	0.55
7	Adult: If someone were to mug me and rob me, I would be more in favor of helping him than punishing him.	Below criteria (Sch.)					
	Adolesc: If someone, for example, mugs me and robs me, I would like it better if they got help instead of being punished for it.	4/10 DM 9/10 CAT					
28	Adult: I often find myself being very nice to people who by all rights I should be angry at.	Below criteria (Sch.)					
	Adolesc: I've realized many times that I'm very nice to people who I should be perfectly angry at.	7/10 DM−9/10 CAT	0.31^***^	0.14^**^	0.18^***^		
✓	**10. Undoing**					0.29^***^	0.64
32	Adult: After I fight for my rights, I tend to apologize for my assertiveness.	2/8 (Chabrol)					
	Adolesc: When I've had to clearly stand up for my needs or rights, I tend to apologize for my harshness afterwards.	7/10 DM−10/10 CAT	0.25^***^		0.15^**^		
40	Adult: When I have an aggressive-angry thought, I feel the need to do something to compensate for it.	✓					
	Adolesc: Whenever I had an aggressive thought, I immediately feel the need to do something to make up for it.	6/10 DM−9/10 CAT	0.11^*^				
	
✓	11. Autistic Fantasy					0.53^***^	0.53
14	Adult: I get more satisfaction from my fantasies than from my real life.	✓					
	Adolesc: I experience more fulfillment and joy in my fantasies than in reality	9/10 DM−6/10 CAT	0.45^***^	0.33^***^	0.10^*^		
17	Adult: I work more things out in my daydreams than in my real life.	✓					
	Adolesc: I often live my life in daydreams.	2/10 DM−5/10 CAT	0.41^***^	0.25^***^	0.19^***^		
X	12. Denial					0.09	0.43
8	Adult: People say I often bury my head in the sand. They say, I tend to ignore unpleasant facts as if they didn't exist.	✓					
	Adolesc: I've been told many times that Iwould ignore unpleasant facts as if they didn't exist.	7/10 DM−2/10 CAT	0.31^***^	0.15^**^	0.14^**^		
18	Adult: I fear almost nothing.	✓					
	Adolesc: I am not afraid of whatever.	6/10 DM−5/10 CAT		−0.11^*^	−0.10^*^		
X	13. Dissociation					0.23^***^	0.03
9	Adult: I don't pay attention to danger as if I was invulnerable.	1/8 (Chabrol), Elimin. (Sch.)					
	Adolesc: I rather don't pay attention to dangers because I have a strong and secure feeling that nothing will happen to me.	0/10 DM− 2/10CAT					
15	Adult: I've special talents that allow me to go through life with no problems.	0/8 (Chabrol), Elimin. (Sch.)					
	Adolesc: I feel I can go through life without much trouble because I am gifted or protected in a special way.	0/10 DM−3/10 CAT	−0.24^***^	−0.25^***^	−0.14^**^		
✓	**14. Isolation**					0.31^***^	0.46
34	Adult: I'm often told that I don't show my feelings.	3/8 (Chabrol)					
	Adolesc: I've been told many times that I don't show my feelings or that I have a ”poker face.	7/10 DM−1/10 CAT	0.34^***^	0.21^***^	0.13^*^		
37	Adult: Often I don't feel much in a situation where strong feelings arise in others.	✓					
	Adolesc: Most of the time, I don't feel very much in “emotional” situations that would bring up strong feelings in others	6/10 DM−4/10 CAT	0.23^***^	0.16^***^			
X	15. Deevaluation					−0.05	0.58
10	Adult: I pride myself on my ability to cut people down to size.	0/8 (Chabrol), Elimin. (Sch.)					
	Adolesc: I am able to really take someone down when I think it is appropriate.	4/10 DM−6/10 CAT	0.12^*^				
13	Adult: am very restrained when I approach other people.	0/8 (Chabrol), Elimin. (Sch.)					
	Adolesc: I am a very inhibited person.	1/10 DM−2/10 CAT	0.40^***^	0.18^***^			
X	16. Displacement					0.13^**^	0.57
31	Adult: Doctors never really understand what is wrong with me.	0/8 (Chabrol), Elimin. (Sch.)					
	Adolesc: Even when I go to doctors, their advice or therapies do not help me.	0/10 DM−5/10 CAT	0.45^***^	0.29^***^	0.16^**^		
33	Adult: When I'm depressed or anxious, eating makes me feel better.	1/8 (Chabrol), Elimin. (Sch.)					
	Adolesc: When I'm sad or anxious, I start eating.	5/10 DM−3/10 CAT	0.23^***^	0.19^***^	0.17^***^		
X	17. Passive Aggression					0.15^**^	0.66
23	Adult: When my boss annoys and rebukes me, I make a mistake in my work or work slower to get back at him.	✓					
	Adolesc: If my teacher or supervisor has annoyed me, I may be more likely to make mistakes or work more slowly, like as “compensatory justice”.	7/10 DM−1/10 CAT	17^***^				
36	Adult: No matter how much I describe my concerns, I never get a reasonable answer.	3/8 (Chabrol)					
	Adolesc: Even when I can accurately describe my concerns, I usually feel like I don't get a reasonable or satisfying answer from the person I'm talking to	0/10 DM−4/10 CAT	0.54^***^	0.29^***^	0.22^***^		
✓	**18. Projection**					0.41^***^	0.70
6	Adult:Everyone is against me.	2/8 (Chabrol)					
	Adolesc: People often behave unfairly toward me or treat me badly.	7/10 DM−1/10 CAT	0.52^***^	0.28^***^	0.23^***^		
29	Adult: I always get treated unfairly.	3/8 (Chabrol)					
	Adolesc: I know from experience that somehow there will always be obstacles or arguments for me, as if “kick me” were written on my forehead.	7/10 DM−4/10 CAT	0.53^***^	0.33^***^	0.21^***^		
✓	**19. Somatization**					0.48^***^	0.54
12	Adult: I get physically ill when things aren't going well for me.	✓					
	Adolesc: I tend to get physically ill when things are not going well for me.	10/10 DM−4/10 CAT	0.40^***^	0.22^***^	0.25^***^		
27	Adult: I get a headache when I have to do something I don't like.	✓					
	Adolesc: I automatically get a headache (or similar) when I have to do things I don't like.	10/10 DM−4/10 CAT	0.44^***^	0.22^***^	0.28^***^		
✓	**20. Splitting**					0.20^***^	0.54
19	Adult: same	✓					
	Adolesc: Sometimes I think I am good like an angel and other times I think I am bad and evil like a devil.	7/10 DM−10/10 CAT	0.42^***^	0.21^***^	0.20^***^		
22	Adult: As far as I'm concerned, people are either good or bad.	✓					
	Adolesc: I am convinced that people are either good or bad.	9/10 DM−10/10 CAT	0.15^**^		0.13^*^		

*Defense mechanism with both items was X = not selected or ✓ = selected for the final test version*

*Adult = english translation of the item formulations in DSQ 40 German version (28) Adolesc. = age-adapted formulations of the items for adolescents*

*DM = Defense Mechanism – CAT = Defense Category*

*✓= no problems with face validity were reported in Chabrol et al. (29) or Schauenburg et al. (28)*

*e.g., 5/10 = number of correct classifications in relation to the number of experts*.

*Structure = OPD-CA2-SQ total score of structural impairment; Depress = PHQ-D depression score; Somat = PHQ-D somatic symptoms score*

r_inter_ = intercorrelation of the two items assigned to the same defense mechanism; f_xy_ = factor loading on the theroetically assigned factor; ad = adaptiv; neur = neurotic; mal = maladaptive; red = below criteria. significance p* = 5%,

** *= 1%, and*

*** *= 0.1% level*.

All ten items to assess adaptive DM in adolescence showed a good (70% correct attributions, reached by 4/10 items) or sufficient (> 40% correct attributions, 6/10 items) face validity in the expert rating. This matched with the results for the original items for adults reported in Chabrol et al. (29) and Schauenburg et al. (28). However, both items to represent the mechanism “Rationalizatio” (items 5, 16) and one item of “Suppression” (item 25) had been misjudged as neurotic by the majority of the experts (70–80%) and were, thus, regarded as maladaptive instead of adaptive.

From the ten items representing neurotic DM, only one item showed insufficient face validity (< 40% correct attributions): item 39 from the mechanism “Pseudoaltruism” was only correctly attributed by 30% of the experts, 60% even attributed it to the category adaptive instead of maladaptive. This matched with the face validity in the adult samples, Chabrol et al. (29) as well as Schauenburg et al. (28) had eliminated this item because of weak face validity. From the remaining nine items, five items showed good and four items showed sufficient face validity, while both items of the mechanism “Reactive Formation” (items 7, 28) and one item of “Undoing” (item 32) showed improved coefficients compared to the study on the adult formulations. Concerning the higher-level category, both items to represent the mechanism “Acting Out” (items 11, 20) had been misjugded as maladaptive instead of neurotic, i.e. were attributed to a higher level of immaturity.

From the twenty items representing maladaptive DM, only five items showed a good face validity and eight items showed a sufficient face validity in the expert rating. Highly similar to the adult version, both items of the mechanism “Dissociation” (items 9, 15) had never been attributed correctly (0/10 experts). For both the meachanisms “Deevaluation” and “Displacement”, one of the each two items showed substantially improved face validity, going together with strong reformulations of the items in order to suit adolescent self-report. Likewise, both items of the mechanism “Projection” (items 6, 29) showed improved face validity compared to the Chabrol et al. (29) study, going along with strong reformulations.

### Reliability and Construct Validity of the Items in the Adolescent Sample and Final Item Selection

In order to evaluate the total psychometric quality of an item and decide on its final rejection or selection, all four established quality criteria were evaluated in a synopsis. Ideally, an item should show both good face validity in the expert test and a highly significant and substantial intercorrelation with the partner item and a significant correlation with an external criterion for psychopathology in the assessments with adolescents in the school and clinic study. In addition, the DM formed by using the each item should show a substantial factor loading on the theoretically assigned factor (adaptive, neurotic, maladaptive). Table 2 shows all coefficients for all items and defense mechanisms. In the following, summarized results per defense category are reported.

From the ten items representing adaptive DM, which are assumed to speak for healthy development, only five items showed significant negative correlations with at least one score denoting pathological development, i.e., impaired structure [OPD-CA2-SQ; (12)], depression [PHQ-D; (38)], or somatic symptoms [PHQ-D; (38)]. Four of five item pairs showed sufficient intercorrelation (except Anticipation), four of five item pairs produced a sufficient loading of the DM on the assigned higher-order factor adaptive (except Rationalization).

From the ten items to assess neurotic DM in adolescence, six showed significant positive correlations with the external pathological variables. Only one of five item pairs met the criteria for sufficient intercorrelation. However, the remaining four at least reached highly significant intercorrelations above 0.20. Four of the five builded DM showed a sufficient loading on the assigned higher-order factor neurotic (except Acting Out).

From the twenty items representing maladaptive DM, seventeen showed positive correlations with psychopathology, most of them even highly significant. However, two items showed negative correlations with impaired structure, depression and/or somatic symptoms and, thus, do not match the attempt to capture a dysfunctional construct. Only four of ten item pairs met the criteria for sufficient intercorrelation. Two more item pairs reached at least highly significant intercorrelations above 0.20. However, the remaining four item pairs showed insufficient intercorrelations between −0.05 and 0.15. In contrast, nine of the ten builded DM showed a sufficient loading on the assigned higher-order factor maladaptive (except Dissociation).

Altogether, only for two DM and their items all criteria were met perfectly (Humor, Somatization). Thus, for the selection of the items to establish the final version of the test, we allowed minor shortfalls in one of the criteria if all other criteria were met (see Table 2). For example, two items (27, 40) of the category adaptive showed a face validity in the expert test below the criteria of 40% correct assingnment, but only either in the concrete DM or in the higher-order defense category, while all other criteria concerning item intercorrelation, relation to pathology and factor loading were met. Similarly in the category neurotic, three intercorrelations of item-pairs to represent a joint DM were highly significant but slightly below the criteria > 0.30 (0.24, 0.24, 0.29) while all other criteria were met.

The result was a final version *DSQ-22-A* for adolescents, capturing 11 DM with 22 Items with sufficient psychometric quality.

### Scale Reliability, Construct and Clinical Validity of the Selected Version DSQ-22-A

The finally selected version *DSQ-22-A* contains each three DM to cover adaptive and neurotic defenses and five DM to cover maladaptive defenses. In an exploratory factor analysis matching the approaches taken with the adult versions (PCA, Varimax, restricted to 3 components), the 11 DM explained 48,4% of the variance, the factor loadings matched the theoretically assigned scale structure of adaptive, neurotic and maladaptive to a great extent (see Table 3).

**Table 3:** *DSQ-22-A* factor loadings >0.30 of the selected 11 defense mechanisms.

	**1 = adaptive**	**2 = neurotic**	**3 = maladaptive**
Humor	**0.61**		
Sublimation	**0.66**		
Suppression	**0.75**		
Idealization		**0.72**	
Reactive Formation		**0.56**	0.32
Undoing		**0.71**	
Autistic Fantasy			**0.73**
Affect Isolation	0.42		**0.60**
Projection			**0.68**
Somatization		0.36	**0.51**
Splitting		0.39	**0.33**

*Bold: considered defense mechanisms for the respective defense category*.

The higher-order scales adaptive, neurotic and maladaptive – as a sum of the each assigned items – showed sufficient scale reliabilities Cronbach's Alpha with 0.63, 0.56 and 0.68, respectively (see Table 4). No significant score differences were obtained according to sex in the scales neurotic and maladaptive. For the scale adaptive the differences where significant on 1% level but with only a small effect size, thus, sex was not incorporated as potential factor in the further analyses. In terms of a reasonable convergent and clinical validity, the defense scale adaptive showed negative correlations with the external variables denoting psychopathology “impaired structure” [OPD-CA2-SQ; (12)], “depression” [PHQ-D; (38)] and “somatic symptoms” [PHQ-D; (38)] in the mixed sample from schools and clinics of *n* = 396 adolescents. The correlations were highly significant but reached only small effect sizes. Similarly, the defense scale neurotic showed positive significant correlations with pathology but only between 0.10 and 0.25. In contrast, the defense scale maladaptive showed not only highly significant but also remarkable correlations with psychopathology, especially with impaired structural impairment (0.75) assessed using the psychodynamic OPD-CA-2 concept [OPD-CA2-SQ; (12)]. When contrasting the scale scores between the school sample (*n* = 261) and the clinic sample of patients (*n* = 135), the defense scale maladaptive showed the highest clinical validity in terms of differentiating the two given groups highly significant and with a large effect size of *d* = 0.9 standard deviations. The defense scale adaptive was able to discriminate between the school and clinical sample highly significant with a medium effect size, while the defense scale neurotic showed no sufficient result in this analysis on clinical validity.

**Table 4:** Basic psychometric properties of the *DSQ-22-A* scales, reliabilities Cronbach's Alpha (α), Pearson correlation (*r*) with related scales, effect size Cohen's d of group differences in ANOVA.

			**Correlation** ***r*** **with related concepts**			**Difference according to PHQ-D health status**				
**DSQ-22-A Scale**	**No items**	**α**	**Structure**	**Depress**	**Somat**	**Healthy *n* = 261**	**Impaired *n* = 135**			
						***M (SD)***	***M (SD)***	***F***	***p***	***d***
adaptive	6	0.63	−0.28^***^	−0.25^***^	−0.14^**^	31.1 (7.6)	27.2 (8.8)	21.888	0.000^***^	0.5
neurotic	6	0.56	0.25^***^	0.10^*^	0.14^**^	21.2 (8.7)	23,0 (8.4)	3.618	0.058	0.2
maladaptive	10	0.68	0.75^***^	0.44^***^	0.34^***^	22.2 (10.4)	32,8 (13.8)	73.707	0.000^***^	0.9

*n, sample size; M, Mean; SD, standard deviation; F, statistical test variable.*

*effect size: r > 0.10 small, > 0.30 medium, > 0.50 strong; effect size: d > 0.20 small, >0.50 medium, >0.80 large.*

significance p* = 5%,

^**^ = 1%,

^***^ = 0.1% level.

To analyze the covariation between the defense scales and the psychodynamic concept of personality structure assessed with the OPD-CA2-SQ (12) in more detail, correlations were calculated for all primary scales and subscales of the OPD-CA-2 (7) concept (see Table 5). The result pattern was stable on primary and subscale level: adaptive defense correlated negative with the scales denoting impairment in structure, whereas neurotic and maladaptive defenses showed positive correlations with impairments. However, on subscale level interesting differences were obtained. For example, adaptive DM showed the least covariation with the structural concepts of coherence (−0.06), emotional contact (−0.07), empathy (−0.07) and attachment relationships (−0.05), originating from different primary scales. Similarly, the correlational pattern showed a range from 0.00 to 0.27 between the defense category neurotic and the subscale level of structural impairment. The defense category maladaptive consistently showed highly significant correlations with all subscales that mostly reached large effect sizes.

**Table 5:** Pearson correlation (*r*) of the *DSQ-22-A* scales with all scales and subscales of OPD-CA2-SQ.

	***DSQ-22-A*** **scales**		
**OPD-CA2-SQ scales**	**adaptive**	**neurotic**	**maladaptive**
**Total score: impairment in personality structure**	−0.28^***^	0.25^***^	0.75^***^
1. Control	−0.31^***^	0.24^***^	0.67^***^
1.1 Impulse control	−0.18^***^	0.17^***^	0.53^***^
1.2 Affect tolerance	−0.34^***^	0.26^***^	0.55^***^
1.3 Conscience formation	−0.21^***^	0.13^**^	0.50^***^
1.4 Self-worth regulation	−0.27^***^	0.21^***^	0.60^***^
2. Identity	−0.22^***^	0.24^***^	0.72^***^
2.1 Coherence	−0.06	0.18^***^	0.60^***^
2.2 Self-experience	−0.26^***^	0.20^***^	0.58^***^
2.3 Self-object differentiation	−0.22^***^	0.24^***^	0.57^***^
2.4 Object experience	−0.14^**^	0.24^***^	0.55^***^
2.5 Belonging	−0.17^***^	0.09	0.54^***^
3. Interpersonality	−0.24^***^	0.23^***^	0.72^***^
3.1 Fantasies	−0.34^***^	0.20^***^	0.57^***^
3.2 Emotional contact	−0.07	−0.00	0.45^***^
3.3 Reciprocity	−0.12^*^	0.10^*^	0.57^***^
3.4 Affective experience	−0.18^***^	0.24^***^	0.58^***^
3.5 Empathy	−0.07	0.21^***^	0.59^***^
3.6 Ability to detach oneself	−0.25^***^	0.26^***^	0.34^***^
4. Attachment	−0.32^***^	0.21^***^	0.67^***^
4.1 Access to attachm. represent.	−0.22^***^	0.16^***^	0.58^***^
4.2 Secure internal basis	−0.33^***^	0.27^***^	0.58^***^
4.3 Capacity to be alone	−0.33^***^	0.15^**^	0.30^***^
4.4 Use of attachment relationships	−0.05	0.02	0.47^***^

*Effect size: r >0.10 small, >0.30 medium, >0.50 strong;*

significance p^*^ = 5%,

^**^ = 1%,

^***^ = 0.1% level.

## Discussion

The attempt to adapt the traditional concept of DM to adolescents as young as 12 years of age in self-report with similar psychometric properties compared to adults can be considered successful. This applies to both the face validity in an expert test as to the reliability of the items and higher-order factors and their clear references to psychopathology. Based on an age-adapted version of the DSQ-40 with simplified formulations, a reliable and valid test version for adolescents with 22 items (*DSQ-22-A*) assessing 11 DM could be built as a result of a thorough empirical item analyses and selection.

The expert test to evaluate the *face validity* of the items showed very similar results for the German adolescent version compared to the results for the adult versions in English (29) and in German language (28), to which we referred in detail. Out of the 40 items of the research version that had been reformulated to be easier to understand for adolescents, only seven items showed a substantially different result concerning being correctly attributed to the theoretically assigned DM. Of those, six items showed improved face validity. Thus, a little improvement in face validity could be reached by using simplified formulations. However, altogether only fourteen of the 40 items showed a very good face validity with at least 70% correct attributions by the experts. This consistency in result patterns concerning face validity across different languages and age groups could be taken as an opportunity to intensively re-discuss the theoretical foundations of the DSQ questionnaire family.

Based on an assessment at 396 adolescents we analyzed further psychometric properties in detail to perform an empirical *item selection* considering several coefficients in a synopsis: (a) good face validity in the expert test with at least 4/10 correct assignments of items to DM or defense category by the experts, (b) highly significant and substantial intercorrelation with the partner item, (c) significant correlation with an external criterion for psychopathology and d) substantial and highest factor loading > 0.30 on the theoretically assigned factor (adaptive, neurotic, maladaptive). Based on this, a total of 22 items forming 11 DM with sufficient psychometric quality could be selected.

Giving up the 2-item-method per DM, more items could have been selected in total to build the higher-order scales adaptive, neurotic and maladaptive. At least six items with sufficient psychometric properties, when evaluated without regard to the intercorrelation with the paired item, could have been additionally integrated. Thus, six further DM could have been represented with at least one item in the final assessment tool (Anticipation, Pseudoaltruism, Denial, Deevaluation, Displacement, Passive Aggression) that now are eliminated. Vice versa, already in the original version of Andrews et al. (25), according to Schauenburg et al. (28), some items showed insufficient content validity but were kept in the test just in order to match the 2-item-method per DM, weakening the reliability of the scales in total.

In line with Schauenburg et al. (28) and Chabrol et al. (29) we tested the adequateness of a 3**-***factor solution* as postulated for the DSQ-40 by the original authors. The 11 DM – build of the selected item pairs – showed factor loadings that matched the theoretically assigned scale structure of adaptive, neurotic and maladaptive to a great extent and met the criteria (> 0.30). Except one (Splitting with 0.33), all DM showed their highest loading (between 0.51 and 0.71) at the assigned factor, together 48.4% of the variance was explained.

The *scale reliabilities* of the finally selected *DSQ-22 A* can be considered as adequate to good, compared to the other DSQ versions also using the 2-item-method for building the single DM (21, 25, 36). For the defense category adaptive (six Items) we obtained a Cronbachs Alpha of 0.63, for neurotic (six Items) of 0.56 and for maladaptive (ten items) of 0.68. Although it is possible to consider using Spearman-Brown instead of Cronbach's alpha to calculate the scale reliabilities (41) for those 2-item-method DM, we kept to the methods used by other authors in order to compare the results directly. Other studies also found the highest scale reliability for the maladaptive defense category, followed by the adaptive and neurotic defense category. For the maladaptive defense category, the scale reliability of the *DSQ-22-A* is slightly below the values of other studies [(25): 0.80; (21): 0.78; (36): 0.75], however, meeting the criteria. Concerning the neurotic defense category, other studies also only achieved values < 0.60 [(25): 0.58; (21): 0.60; (36): 0.60]. For the adaptive defense category, the *DSQ-22-A* actually showed similar coefficients compared to the other studies on adolescent samples [(21): 0.62; (36): 0.58]. A modified version of the DSQ for adolescents with a different number of items per DM (31) did show mostly lower scale reliabilities compared to the *DSQ-22-A* (mature: 0.52, neurotic: not reported, immature: 0.81).

*Convergent validity* of the *DSQ-22-A* could be shown by significant correlations between the defense scales and related scales of psychopathology in terms of “impaired structure” [OPD-CA2-SQ; (12)], “depression” and “somatic symptoms” [PHQ-D; (38)]. The defense categories correlated with impairments in personality structure according to the theoretical expectation (the adaptive defense category correlated negative with impairments, the neurotic and maladaptive defense categories correlated positive). Especially the maladaptive defense category showed high relations to the three measures of psychopathology, highest with impaired structure (0.75, 0.44, 0.34). This is in line with theory, as it is assumed that that a low integrated structural level is associated with the use of immature DM (7). Also these findings are in concordance with the results by Ruuttu et al. (21), who found the strongest associations between the immature defense category and psychopathology (psychiatric symptoms: 0.65; adaptation: −0.54), whereas the associations to the other defense catgories showed only small to medium effect sizes. Compared to the results of Giovazolias et al. (36), who used the DSQ-40 in their adolescent sample and found only small effect sizes for convergent validity, the *DSQ-22-A* showed medium to strong effect sizes for the defense scale maladaptive in the adolescent sample.

To our knowledge, this is the first study to use personality structure as an external criterion for evaluating the convergent validity of a DSQ version. Such an analysis was urgently needed against the background of the described close connection of the psychodynamic construct “personality structure” and the concept “personality functioning” and its relevance in the new dimensional diagnosis of PD [ICD-11; (3)]. Thus, we analyzed the correlational patterns in detail not only on total scale but also on primary and subscale level. Interestingly, the DM of the adaptive and neurotic defense categories showed very diverse correlational pattern with the subdimensions of personality structure. For example, it seems controversial in terms of content that the adaptive defense category is little (small effect sizes) correlated with empathy (−0.07) and emotional contact (−0.12) (both subdimensions of the dimension interpersonality) and use of attachment relationship (−0. 05) (subdimension of the dimension attachment), but more clearly (medium effect sizes) with affect tolerance (−0.34) (subdimension of the dimension control), fantasies (−0.34) (subdimension of the dimension interpersonality) as well as with secure internal basis (−0.33) and capacity to be alone (−0.33) (both subdimensions of the dimension attachment). These detailed covariations might inform the discussion of a revised formulation of some items to represent the core of healthy vs. problematic DM. However, the maladaptive defense category consistently showed highly significant correlations with mostly large effect sizes with all subscales of personality structure. This indicates a high covariation between the two psychodynamic concepts in terms of impaired personality functioning.

Regarding the *convergent validity*, the maladaptive defense category in particular was able to differentiate highly significant between the school and clinical sample with a large effect size of *d* = 0.9 standard deviations. The adaptive defense category discriminated between the both groups highly significant with a medium effect size of *d* = 0.5 standard deviations, while the neurotic defense category showed no sufficient clinical validity. This finding is consistent with similar studies, e.g., in the original publication by Andrews et al. (25) the mature and immature defense categories showed higher effect sizes than the neurotic defense category according the discrimination between a clinical and a healthy sample. Studies focusing on adolescent samples also reported less differentiation between clinical and school samples by the neurotic defense category compared to the other defense categories (21, 31). Giovazolias et al. (36) also showed in a logistic regression analysis that only the immature and mature defense categories predicted wellbeing, bullying behavior and victimization in a nonclinical sample in a statistically significant manner, albeit with small effect sizes.

There are several considerations how psychometric properties of assessment tools to capture DM might be improved in general. First, for those DM with consistently weak face validity, completey new formulations might be discussed. E.g., the two items representing the clinically important DM Dissociation do not seem to capture the theoretically described content of this aspect at all (in the adult as well as in the adolescent version). Likewise, all DM that were rejected because of their insufficient face validity (e.g., Devaluation, Displacement, Passive Aggression) might be openly discussed and reformulated in a way that would fit better to the underlying descriptions focusing related pathological behavior. Second, a reasonable consideration for improving the psychometric properties of the DSQ family of questionnaires might be whether the 2-item method should be abandoned. It could be analyzed to what extent an increase in the number of items per DM would improve the questionnaire. In this context it would also be reasonable to consider whether only the power DM with good reliability should be included and operationalized at all in order to provide a shorter questionnaire. Most importantly, it would be reasonable to make a restriction to those DM that are clearly related to psychopathology. Thus, it should be considered to omit the neurotic defense category, as this showed the poorest results concerning reliability and especially clinical validity. This would lead to retaining only the adaptive and the maladaptive category. In principle, it would be possible to derive a different version of the DSQ-A from the current study that contains all reliable and valid items of only these categories. However, with the *DSQ-22-A* we provide a youth-specific version of the DSQ with sufficient psychometric quality.

Some *limitations* should be noted with respect to the present study. First, it is a cross-sectional study. In the future, longitudinal studies should be performed to test clinical validity in terms of predictability of specific symptomatology, especially in interaction with impairments in personality functioning in adolescents. Moreover, the present study used a German sample. It is possible that culture-specific aspects influence the applicability of certain DM. However, this seems unlikely given the numerous translations of the DSQ with very similar result patterns. Since the clinical group consists of adolescents with a variety of psychiatric symptomatology, homogeneity is limited and generalization to specific groups of patients is not possible. On the other hand, mixed forms and multiple diagnoses in relation to mental disorders correspond to clinical reality [e.g., (42)]. Moreover, it must be taken into account that both groups in the present study included more girls than boys. In addition, there were significant age differences between the two groups, so that representativeness might be limited. Future studies might therefore cross-validate the obtained score levels, for example by assign all adolescents of a representative school. Finally, the use of the PHQ-D (38) as a self-assessment tool to assess mental health is not optimal but was chosen due to economic reasons. Future studies should include, at least for the clinical sample, the use of clinical structured interviews as the gold standard of clinical diagnosis (38). However, the PHQ-D (38) already proved its worth in other studies concerning the screening of mental disorders in adolescents [e.g., (42)].

Overall, the *DSQ-22-A* comprises reliable and valid item pairs and shows adequate covariations with psychopathology in adolescents comparable to the DSQ-40 (25) or the DSQ of Schauenburg et al. (28) for adults. In its current design, it can be used in adolescent samples in German-speaking countries with preliminary population norms. In the light of the new diagnostic guidelines for PD in the upcoming ICD-11, following a dimensional severity concept which allows the assessment of several domains of personality functioning already from early adolescence, the assessment of DM may inform clinical decision making and therapy planning. Especially immature defense mechanisms assessed with the *DSQ-22-A* may help to understand specific aspects of impaired personality structure which can be regarded as “the psychodynamic twin” of the concept of personality functioning.

All 11 DM assessed by the DSQ-22-A can be used for research on defense mechanisms with adolescent samples from 12 years up. For future developments, however, the basic operationalization's and the number of relevant DM should be discussed internationally. The detailed information regarding the psychometric properties of the item pool used for building the *DSQ-20-A* in this publication might be a good basis for this purpose.

## Data Availability Statement

The datasets presented in this article are not readily available because the participants of this study did not agree for their data to be shared publicly. Requests to access the datasets should be directed to lea.sarrar@medicalschool-berlin.de.

## Ethics Statement

The study involved human participants and was reviewed and approved by Research Ethic Board at the MSB Medical School Berlin. Written informed consent to participate in this study was provided by the participants' legal guardian/next of kin.

## Author Contributions

LS and KG contributed to conception and design of the study and wrote sections of the manuscript. LS organized the database. KG performed the statistical analysis. All authors contributed to manuscript revision, read, and approved the submitted version.

## Conflict of Interest

The authors declare that the research was conducted in the absence of any commercial or financial relationships that could be construed as a potential conflict of interest.

## Publisher's Note

All claims expressed in this article are solely those of the authors and do not necessarily represent those of their affiliated organizations, or those of the publisher, the editors and the reviewers. Any product that may be evaluated in this article, or claim that may be made by its manufacturer, is not guaranteed or endorsed by the publisher.
