# Supplementary material for: Defense Mechanisms Reloaded in the Light of Impaired Personality Functioning: An Attempt of Clarification and Simplification Resulting in the DSQ-22-A for Adolescents
Source: Front Psychiatry. 2022 May 27;13:866837. doi: 10.3389/fpsyt.2022.866837 (PMC9198968; doi:10.3389/fpsyt.2022.866837)
Supplement: Supplementary file 1 [file Data_Sheet_1.PDF]

Supplement. Selection of DSQ studies

study	DSQ version	DM (*n*)	*n*	defense categories and *n* of included defenses (if stated); reliability of the defense categories (if stated)
Bond, Gardner, Christian & Sigal (1983)	81	24	209	4 defense categories (no statement of α)<br>defense style 4 (adaptive): 3 defenses<br>defense style 3 (self-sacrificing): 2 defenses<br>defense style 2 (image-distorting): 3 defenses<br>defense style 1 (maladaptive action patterns): 6 defenses
Vaillant, Bond & Vaillant (1986)	67	15	131	3 defense categories (no statement of α)<br>mature: 4 defenses<br>neurotic: 3 defenses<br>immature: 8 defenses
Andrews, Pollack & Stewart (1989)	82/36	20	413	3 defense categories (no statement of α)<br>mature: 4 defenes (both versions)<br>neurotic: 4 defenses (82-version)/3 defenses (36-version)<br>immature: 12 defenses (82-version)/10 defenses (36-version)
Andrews, Singh & Bond (1993)	40	20	712	3 defense categories<br>mature: 4 defenses; α = .68<br>neurotic: 4 defeneses; α = .58<br>immature: 12 defenses; α = .80
**Steiner & Feldman (1995)**	78	21	272	3 defense categories<br>mature: 5 defenses; *α* = .52<br>neurotic: 2 defenses; *α* = /<br>immature: 12 defenses; *α* = .81
**Erickson, Feldman & Steiner (1996)**	78	19	140	3 defense categories (no statement of α)<br>mature: 5 defenses<br>prosocial: 2 defenses<br>immature: 12 defenses
**Feldman, Araujo & Steiner (1996)**	59	19	693	3 defense categories<br>mature: 5 defenses; α = .52<br>prosocial: 2 defenses; α = /<br>immature: 12 defenses; α = .81
**Nasserbakht, Araujo & Steiner (1996)**	78	26	737	4 defense categories (no statement of α)<br>factor I: 10 defenses<br>factor II: 6 defenses<br>factor III: 2 defenses<br>factor IV: 1 defense
**Erickson, Feldman & Steiner (1997)**	59	19	81	3 defense categories (no statement of α)<br>mature: 5 defenses<br>prosocial: 2 defenses<br>immature: 12 defenses
Bonsack, Despland & Spagnoli (1998)<br><br>(French translation)	88	24	222	4 defense categories<br>adaptive: 2 defenses; α = /<br>minor disorder: 5 defenses; α = .59<br>neurotic: 3 defenses; α = .41<br>maladaptiv: 7 defenses; α = .71
**Steiner, Araujo & Koopmann (2001)**	71 (REM-71)	21	1875	2 defense categories<br>factor 1: 14 defenses; α = .84<br>factor 2: 7 defenses; α = .68

Supplement. Selection of DSQ studies

study	DSQ version	DM (*n*)	*n*	defense categories and included defenses (if stated); reliability of the defense categories (if stated)
San Martini, Roma, Sarti, Lingiardi & Bond (2004)<br><br>(Italian translation)	88	25	628	3 defense categories<br>adaptive: α = .57<br>image-distorting: α = .72<br>maladaptive: α = .85
Chabrol, Rousseau, Rodgers, Callahan, Pirlot & Sztulman (2005)	40	20	/	study on the face validity of the DSQ-40<br>(results suggesting lack in face validity)
**Ruuttu, Pelkonen, Holi, Karlsson, Kiviruusu, Heila, Tuisku, Tuulio-Henriksson, LicPhil & Marttunen (2007)**<br><br>(Finnish translation)	40	20	410	4 defense categories<br>mature: 5 defenses; α = .62<br>neurotic: 4 defenses; α = .60<br>image-disorting: 5 defenses; α = .62<br>immature: 6 defenses; α = .78
Schauenburg, Willenborg, Sammet & Ehrenthal (2007)<br><br>(German translation)	40	20	155	3 defense categories (no statement of *α*)<br>adaptive (mature): 4 defenses<br>intermediate (neurotic): 4 defenses<br>maladaptive (immature): 4 defenses
Thygesen, Drapeau, Trijsburg, Lecours & de Roten (2008)<br><br>(French-DSQ-60 version)	60	30	517	3 defense categories<br>adaptive: 5 defenses; α = .61<br>affect regulating: 4 defenses; α = .72<br>image distorting: 5 defenses; α = .64
Saint-Martin, Valls, Rousseau, Callahan & Chabrol (2013)	28	16	201	5 defense categories<br>mature: 5 defenses; α = .47<br>autistic fantasy: 2 defenses; α = .61<br>neurotic defenses: 3 defenses; α = .42<br>denial: 2 defenses; α = .34<br>immature: 4 defenses; α = .60
**Giovazolias, Karagiannopouloub & Mitsopoulouc (2017)**<br><br>(Greek translation)	40	20	265	4 defense categories<br>mature: 3 defenses; α = .58<br>neurotic: 4 defenses; α = .60<br>image-distorting: 4 defenses; α = .61<br>immature: 7 defenses; α = .75
De Page, van der Heijden, De Weerdt, Egger & Rossi (2018)	42	21	445	3 defense categories<br>mature: 4 defenses; α = .58<br>neurotic: 4 defenses; α = .41<br>immature: 11 defenses; α = .76

Notes: studies sorted by year; bold = studies in children or adolescents; DSQ = Defense Style Questionnaire; DM = defense mechanisms; *n* = number/sample size; α = Cronbach´s Alpha
